# Assessing climate and health curriculum in graduate public health education in the United States

**DOI:** 10.3389/fpubh.2023.1124379

**Published:** 2023-04-17

**Authors:** Mona Arora, Andrew C. Comrie, Kacey E. Ernst

**Affiliations:** ^1^Department of Community, Environment, and Policy, Mel and Enid Zuckerman College of Public Health, University of Arizona, Tucson, AZ, United States; ^2^School of Geography, Development and Environment, College of Social and Behavioral Sciences, University of Arizona, Tucson, AZ, United States; ^3^Department of Epidemiology and Biostatistics, Mel and Enid Zuckerman College of Public Health, University of Arizona, Tucson, AZ, United States

**Keywords:** climate change, curriculum and instruction, training, climate and health education, climate and health

## Abstract

Climate change has been identified as both a challenge and an opportunity for public health. The onus to prepare the next generation of public health practitioners lies heavily on schools and programs of public health. This article (i) assesses the status of climate change and health curricula in accredited schools of public health in the United States and (ii) proposes strategies to better train professionals so they are more informed and prepared to mitigate, manage, and respond to the health impacts of climate change. Course offerings and syllabi listed in online course catalogs from 90 nationally accredited schools of public health were evaluated with the purpose of identifying the extent of climate change education in graduate programs. Only 44 public health institutions were found to offer a climate change related course at the graduate level of education. Of the 103 courses identified, approximately 50% (*n* = 46) are focused on this climate change and health. These courses cover a wide array of topics with an emphasis on conveying fundamental concepts. In-depth assessment revealed a need for integrating learning opportunities that build practical skills useful in a hands-on public health practice environment. This assessment indicates the limited availability of climate-health course offerings available to graduate students in accredited schools. The findings are used to propose an educational framework to integrate climate change into public health curricula. The proposed framework, while rooted in existing directives, adopts a tiered approach that can be readily applied by institutions training the next generation of public health leaders.

## 1. Introduction

Climate change is a complex and present challenge facing current and future generations. Climate change is increasing the frequency, duration, and intensity of climate-driven events (1) posing significant threats to human health and wellbeing. Health impacts include higher incidence of asthma and respiratory disease related to air pollution and allergens; increased deaths due to extreme heat; and, increased risk of waterborne, foodborne, and vector borne disease as a result of higher temperatures (1, 2). The far-reaching and diverse impacts on both environmental and human systems introduce challenges associated with identifying, mitigating, and managing the myriad direct and indirect consequences to health and wellbeing. The opportunity, however, lies in the ability to frame climate change as a public health issue, bringing together sectors and disciplines to adopt a human health-centric, holistic approach to enhancing resilience to climate change.

Governmental and non-governmental organizations including public health agencies at local/municipal, tribal, state, province/district and national levels play a critical role in managing and responding to the health effects of climate change. Although this paper focuses on building public health capacity in the United States, the principles and recommendations described in this paper also apply at an international level. All public health professionals need at least a basic understanding of climate change impacts on human health and wellbeing and how to mitigate, manage and respond to them.

Several national and regional assessments and surveys highlight workforce training needs to prepare public health professionals for understanding and managing the health implications of climate change (3–6). These assessments and surveys describe a pressing need for education and training that builds knowledge of climate science as well as climate-health relationships and illustrates relevance of public health essential functions and principles with climate change impacts (5, 7). In response, professional, non-profit and governmental organizations have initiated webinars, focused trainings, and have integrated climate-health relevant topics in conference agendas and themes. The plethora of resources available on national public health websites as well as the inclusion of climate and health topics on conference agendas (e.g., the 2017 National APHA Conference theme was Climate and Health) indicate climate change is being elevated as a priority in mainstream public health.

Institutions of higher education play a vital role in building future public health professionals’ capacity to understand, manage, and address the health impacts of climate change (8). Higher education institutions can engage students from varied disciplines and programs (both public health and non-public health) fostering values in collaboration and systems thinking that cannot be easily replicated in a workforce environment. As of December 2022, there is currently no direct reference to climate change in the Council on Education for Public Health (CEPH) accreditation criteria; however, there is clear alignment with multiple core competencies at the undergraduate and graduate levels (9, 10). CEPH is the national organization responsible for accreditation of both schools of public health and public health programs while the Association of Schools & Programs of Public Health (ASPPH) is a national organization that advocates for high-quality education standards and comprises of both CEPH-accredited schools and programs of public health as well as those in applicant status for CEPH-accreditation. ASPPH has also released a toolkit in collaboration with the Global Consortium on Climate and Health Education (GCCHE) that “provides practical approaches and tools for integrating climate change and health education into a public health curriculum” (11). Housed in Columbia University, the GCCHE advocates for climate-health education among all health professionals. Public health professional organizations and academic institutions have also begun to emphasize the need and opportunity to enhance the future public health workforce’s understanding and capacity to respond to climate change. Recent initiatives including the release of this toolkit by the ASPPH, in collaboration with the GCCHE, have elevated the need for active involvement and intensive action to prepare the future public health workforce in meeting the climate change challenge. The toolkit is built on the GCCHE core competencies, which are geared toward educating all health professions about climate and health relationships (8).

While several studies highlight the need for climate and health education for the health professions, including public health (8, 12, 13), little is known about the current extent of climate change education in graduate programs of public health in the United States. This study builds on Becker et al. (14) and assesses the extent of climate change and health courses offered at ASPPH institutions of public health in the United States at the graduate level (Master’s and Doctoral degrees) through a broad scoping analysis and reviews of available course syllabi.

## 2. Methods

This study assessed the extent to which ASPPH-accredited colleges of public health integrate climate change into existing graduate curricula by identifying courses listed in online program websites and catalogs. A list of accredited institutions awarding any form of graduate degree in public health (e.g., MPH, MHA, MS, MHS, PhD, ScD, and DrPH) were obtained from the ASPPH website. Each institution’s website, course catalog, and schedule of classes was assessed to identify any course offerings that used a range of terms including “climate,” “climate change,” “global warming,” “environment,” or “environmental change” in the course title and/or description. Results referring to climate in the non-environmental context (e.g., economic, work or cultural climate) were excluded from analysis.

### 2.1. Data collection and analysis

All data were collected between May and July 2018 and maintained in an Excel spreadsheet. Primary syllabus analyses were conducted using NVivo Pro to categorize learning objectives and course deliverables along Bloom’s taxonomy. Course information including title, description, objectives, and delivery (online, hybrid, or face to face), was obtained for each course that was identified. Two levels of analysis were conducted to characterize the integration of climate change into the curricula. Level 1 analysis focused on the extent climate and health topics were addressed in coursework. Level 2 analysis assessed the approach (i.e., learning objectives, content areas) adopted in courses identified in Level 1.

Each course was assigned into one of two categories: Focused, if climate change was deemed to be the focus of the course or Integrated, if climate change was included as one of the topics in the course. Designation into the above categories was determined by a review of the course title and description. If the terms “climate,” “climate change,” “environmental change” or “global warming” were listed in both the title and description then the course was listed under the “Focused” category. If these terms were listed under the course description but not course title and a scan of the course content revealed only a specific lecture on this topic, then the course was designated as “Integrated” referring to the integration of the topic into the course. We distinguished between focused courses and those that integrate climate and health across multiple courses. This provides a more in-depth understanding of the extent to which climate and health is being taught at schools of public health.

### 2.2. Assessment frameworks

Course syllabi were downloaded for any courses that were openly available on the school or program’s website. Contacting authors in a thorough comprehensive and consistent way was not feasible at the time of the study, leading to a focus only on those courses and syllabi easily accessible online. Primary search efforts were focused on current course catalogs and listings; however, any available past course listings were also assessed for this study. Course syllabi and objectives were evaluated using Bloom’s revised taxonomy (15) as an indicator for the degrees of complexity and cognition required in the course. Bloom’s revised taxonomy provides a structure through which instructors can engage students in differing levels of learning. Learning objectives, if provided in the syllabi, were aligned to one of the six taxonomy categories to assess the level at which the climate-health relationship was covered. The verbs used to structure a learning objective were classified into each category to determine the level of learning expected in these courses collectively. Verbs falling under the remember, understand and apply categories were indicative of less complex learning expectations such as defining, classifying, and developing. Verbs associated with analyzing, evaluating, and creating categories were classified as more complex, higher order learning.

## 3. Results

Our course search provided insight into the availability of climate and health courses at schools and programs in public health. Of the public health institutions evaluated in this study, 51% (46 of 90) did not offer any climate change-related course. The remaining 44 institutions were found to offer 103 climate change-related courses of which there were 44.6% (or 46) courses that were *focused* on climate and health while the remaining 57 courses *integrated* this topic into a broader course topic area.

The courses identified were all offered by the public health school or program either currently or within the previous 4 years; they included graduate only and joint undergraduate/graduate courses, and both online and in-person forms of delivery. In addition, 23% of ASPPH schools were found to offer more than 1 course that either focused on or integrated climate change and health.

As each course was identified, further research was conducted to locate and download the course syllabus for further analysis. Syllabi of 46 courses (22 focused, 24 integrated) were readily available for download and review. All graduate course syllabi were reviewed for further analysis, resulting in the exclusion of eight courses that were undergraduate courses, non-public health courses, or climate change associated courses that had incomplete open-access syllabi (6 focused, 2 integrated). Table 1 illustrates the breakdown of courses identified in this study.

**Table 1:** Climate-health course summary.

Number of institutions reviewed	90
*Institutions with zero courses*	46 (51.1%)
*Institutions with at least 1 course*	44 (48.8%)
Number of total courses identified	103
*“Integrated” courses*	57 (55.3%)
*“Focused” courses*	46 (44.7%)
Syllabi available for download and included for analysis	38
*“Integrated” course syllabi*	22 (57.9%)
*“Focused” course syllabi*	16 (42.1%)

*Integrated courses*: Courses were categorized as integrated if “climate change” environmental change,” “global warming,” “environment” or “environmental change” was identified as a course sub-topic or described in a learning objective. The 22 integrated syllabi were reviewed to determine the extent to which climate change, global warming, and/or environmental change were covered in the syllabi.

Although all courses integrated a discussion of climate change as a public health issue, they differed in the scope and context within which the topic was discussed: Fifteen courses were found to describe or list climate change as one of many topics in relation to human health; two courses covered a specific aspect of climate change such as global climate change models and mechanisms of climate change and seven courses discussed the topic in the context of a broader topic (e.g., built environment, deforestation, advocacy and environmental justice, and frameworks to address global challenges).

Among all evaluated courses in this category, only 10 courses included a learning objective in relation to climate change and health.

**Figure 1:**
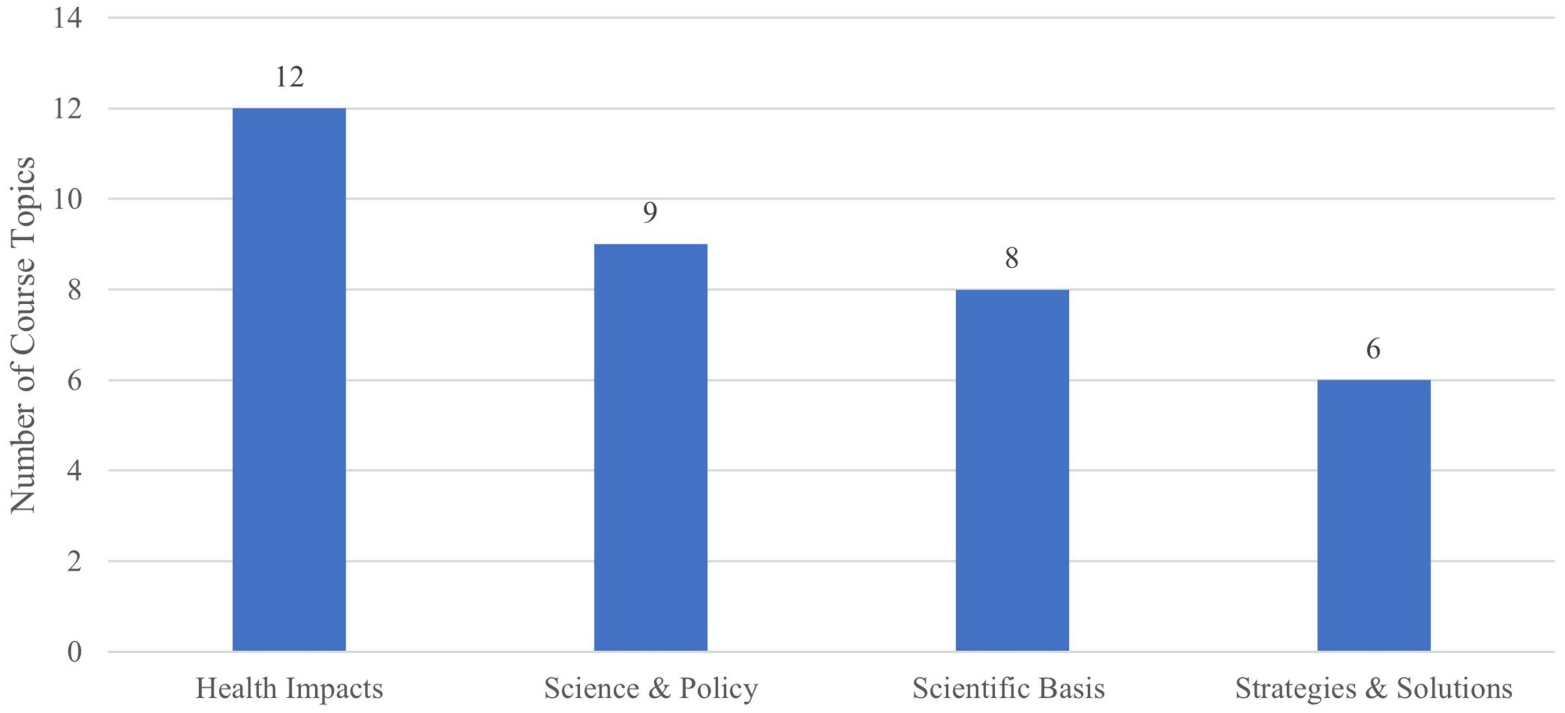
Most commonly taught topics in climate-health courses.

*Focused courses*: Sixteen syllabi for courses specifically focused on “climate change” or “environmental change” and health were analyzed to contextualize how climate change was addressed in curricula. The analysis revealed that 87.5% (or 14) courses were housed in the environmental health science department of the public health program. As illustrated in Figure 1, these courses covered an array of topics.

**Figure 2:**
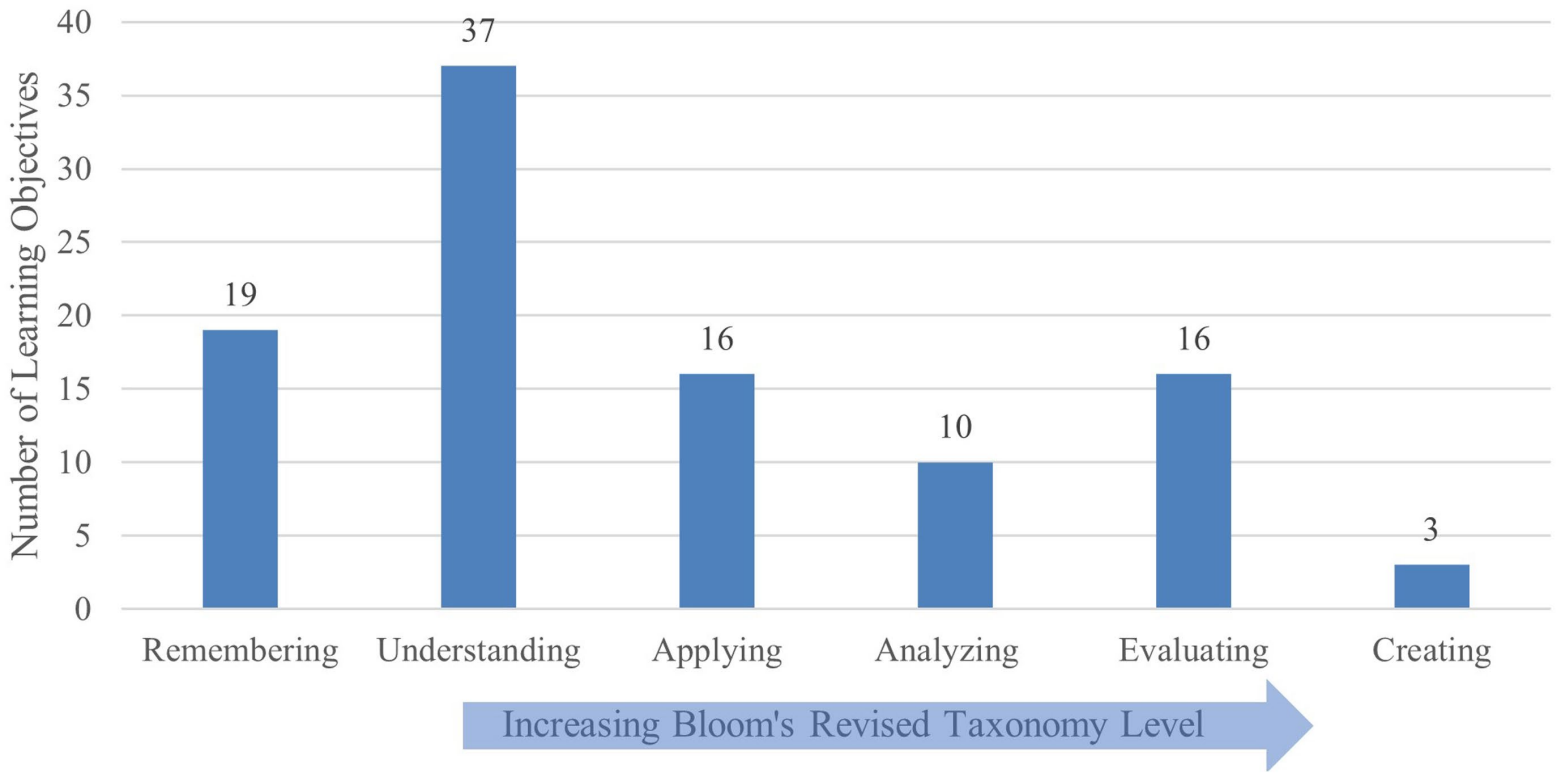
Analysis of focused course learning objectives using revised Bloom’s taxonomy.

Courses also discussed a wide range of relevant issues such as communication, food and water security, and energy and sustainability. A review of the readings list (when available) and course description also indicated the use of both international (i.e., International Panel on Climate Change Report on Global Warming) and national (i.e., United States Global Change Research Program National Climate Assessment) reports to inform students of the current state of knowledge. Figure 2 provides the breakdown of learning objectives across Bloom’s taxonomy.

A review of course syllabi including course evaluation criteria and forms of student assessment indicated term papers, in-class (individual or group) presentations, and exams as preferred methods to evaluate student learning. The evaluated syllabi identified minimal use of case studies and performance on problem-solving, scenario-based activities as assessment formats in these courses. On the other hand, instructors seemed to encourage students to delve into more focused topics of interest through term papers or presentations.

### 3.1. Proposed framework

Drawing on the analysis and findings above, we propose the following 4-tier framework for accredited schools of public health to increase knowledge and awareness as well as skills in managing and addressing the health impacts of climate change. The framework recognizes the challenges (e.g., limited topical expertise, timeframe, staffing, budget, etc.) faced by institutions to create and launch fully functional courses on the topic. Therefore, the proposed framework adopts a tiered approach for easy, staged adoption through which to increase awareness of climate change into public health education. The framework proposes treating climate change as a public health stressor no different than other cross-cutting public health issues (e.g., socio-economic factors) in the curriculum.

The framework proposes that climate and health curricula in public health should:

1. Be rooted in pedagogy that utilizes backwards design methodologies that emphasize skills, learner-centered course design, and integrate active learning approaches.
2. Acknowledge the complexities of graduate in-person (i.e., face to face) and online teaching, including the diverse preparation of students in climate change topics.
3. Provide opportunities for peer-to-peer as well as instructor-led learning.
4. Illustrate the importance of the interdisciplinary nature of the issue and the approaches required to both understand and address it.

We encourage higher education institutions and, in particular, schools and programs of public health to provide students with multiple, consistent opportunities through which to understand the challenges associated with climate change. In the absence of national curricula directives, individual institutions should collaborate with experts and existing communities of practice to develop and share curricula best practices for adoption in their respective public health programs.

At a minimum, we propose that institutions dedicate resources and efforts to integrate a climate change lens into the core public health courses and disciplines (Tier 2-Building knowledge) including biostatistics, environmental health sciences, epidemiology, health policy and management and social and behavioral sciences. This will ensure that students in all public health focus areas not only receive the required fundamental concepts but understand the relevance of this issue to their specific discipline. We acknowledge the time and resource constraints required to undertake the implementation of this goal and accomplish Tier 1 (building awareness) activities through collaboration with academic and field experts to organize guest lectures and forums. In the long-term, public health institutions should strive to complement Tier 1 and 2 educational activities with the development of a course specific to public health and climate change (Tier 3- Enhance knowledge, problem-solving, and critical thinking). Existing toolkits and guidelines can be expanded to include modules and slide decks that cover climate and health basics to support institutions that have limited expertise in attaining at least Tier 1 implementation. Dedicated courses can be co-created with multiple academic (both health and non-health) units to provide a comprehensive, cross-sectoral, and multidisciplinary understanding of the health aspects of the climate change problem. Similarly, once a fundamental basic climate and health course has been established, it is recommended that public health schools and programs consider implementing any special topics courses (Tier 4- Tailored skill building) and/or climate and health concentration areas for developing a skillset in this area. The tiers comprising this framework are summarized in Table 2.

**Table 2:** Proposed climate and health educational framework.

Tier	Objective	Modality	Examples
Tier 1: Building awareness	Provide students with exposure to climate change and health relevant issues.	Single-point lectures and activities to introduce the topic area.	Forums, Brownbag series, Seminars, Colloquiums.
Tier 2: Building knowledge	Ensure that students understand the importance of climate change and its relevance to all public health topic areas.	Climate change lens and case studies integrated into public health core courses.	Module on evaluating existing healthcare policies on how climate and health co-benefits can be achieved.
Tier 3: Enhance knowledge, problem-solving, and critical thinking	Prepare students for assessing and addressing the health impacts of climate change.	Interdisciplinary education opportunities that provide fundamental knowledge and opportunities for application of material to address practical field challenges.	Dedicated course on public health aspects of climate change, Inter-professional education courses.
Tier 4: Tailored skill building	Provide students with the platform to delve into a focused area of study in relation to climate and health.	Specialized course and/or program with a topical focus.	Climate modeling methods course, Special topics course on climate change communication.

## 4. Discussion

Public health practitioners are at the forefront of responding to health outcomes that result from climate change. Whether willing or not, public health is being called upon to take action to prevent, manage, and address the health impacts of climate change (16).

Training the next generation of public health leaders is an initiative that can be addressed by schools and programs in public health. Approximately 50% of ASPPH institutions assessed in this study did not list a course that clearly included climate change content on their program website or course catalog, indicating a need for the development of further structured opportunities in climate and health in graduate public health education. These findings highlight a significant gap in the knowledge and skills provided to the entering public health workforce members, in particular, their awareness and ability to inform public health action and management of climate change impacts.

All focused courses were electives offered by departments and units responsible for the environmental health focus area of public health. We recognize that a certain level of expertise resides within this sub-discipline, but this is done at the cost of overlooking other aspects of the issue (e.g., policy management, health promotion) that may not be as well incorporated into the existing curricula. This assessment corroborates findings from public health professional interviewees that stressed the need to communicate and engage with counterparts in different branches of public health to incorporate the climate change dialogue and inform decisions that have climate and health co-benefits (17).

Current offerings of climate change courses focus on fundamental climate and health relationships and do not cover topics relevant to public health practitioners. Courses cover a wide array of sub-topics referenced in the GCCHE competencies (18), ranging from climate dynamics and environmental drivers of health to human health impacts and public health actions including adaptation, policies, and risk assessments.

The systematic review of the course syllabi implies a tendency to focus on transmitting knowledge of key issues that form the basis of our understanding of climate change and health (i.e., scientific basis, health impacts, and possible solutions) rather than expressing climate change in ways directly relevant to public health practitioners such as public health practice and policy aspects of the issue (e.g., health equity, systems thinking, program development, and planning) (16). While the course content focuses on knowledge, course assessments focus on critical thinking and understanding key concepts as evidenced by research papers and in-depth topic investigations. These findings indicate areas for improvement to optimize the learning opportunities that facilitate practical skill-building and translate knowledge to skills useful in an environment of public health practice.

Graduate public health education needs to prepare students for working effectively in the field. In dealing with the challenge of climate change, graduate public health education should be applied, build a strong foundation in the human-environment dimensions of health, promote interdisciplinary problem-solving and critical thinking, and provide graduates with a set of tools in their toolbox with which to approach problems in the field.

Graduate institutions must meet the needs of future employers. These are the individuals who will find themselves communicating the relevance of a changing climate to their communities, expanding their collaborative networks to include city and county divisions of transportation and urban planning to heighten awareness of the health implications of local decisions; and conducting research to inform the development of tailored, community-based interventions. The proposed framework allows schools and programs to assess their expertise and adopt any combination of the tiers proposed. It is recommended that all institutions implement at least Tier 1 and Tier 2 over the next 5 years. For smaller programs with limited faculty expertise, curricula resources including videos and activities are available through the ASPPH and GCCHE toolkits.

Existing competency frameworks such as the GCCHE provide recommendations on how to achieve foundational knowledge on climate change and health in graduate public health education. Central to existing GCCHE competencies and ASPPH guidelines is the notion of collaborating, communication, and the underlying goal of preparing health workforce (including public health and other health professions) to address the health impacts of climate change. Given the highly integrative and transdisciplinary approach needed to adapt to the global climate crisis, we further recommend that all academic institutions and broad national professional organizations such as GCCHE deepen their collaborations across disciplines and sectors to develop more complex and realistic case studies to enhance training and preparation for the challenges associated with climate change.

### 4.1. Limitations

This study was limited due to inaccessibility of all syllabi and course information on institutional websites. Many institutions did not provide access to the course catalogs on their school or program webpage, potentially resulting in the exclusion of courses that may have been offered in prior years or are offered in a cyclical manner (i.e., every other year). Furthermore, when course listings were provided, course syllabi were often unavailable. Topical seminars offered by schools and programs but not listed with content-specific details were likely missed during the course search process.

Another limitation of this study is that courses outside of the schools of public health were not considered in the analysis unless they were explicitly identified as a required or elective course. Courses may be offered by other departments within the academic institution such as Geography, Environmental Sciences, Natural Resources, or Communications. However, they were not included in this study.

## 5. Conclusion

A review of existing syllabi indicates the need for courses that provide students with meaningful, relevant, and higher order learning on climate change and health. Existing guidelines developed by national organizations such as ASPPH and the GCCHE should inform curricular design. In the absence of climate change in national public health accreditation criteria, these guidelines and calls for action should inform the approach to exposing the public health student to the significance of climate change on public health. However, the inclusion of climate and health in graduate and undergraduate public health program accreditation criteria is long overdue and is needed to ensure a baseline level of competency among graduating, future public health professionals.

Our proposed framework proposes mechanisms to enable public health graduate programs to integrate climate and health education into the existing curricula. The proposed framework allows schools and programs to assess their expertise to adopt any combination of the tiers proposed to provide multiple opportunities for building knowledge and skills among their student body. It is recommended that all institutions, particularly smaller programs with limited expertise, strive to attain Tier 2 and 3 of this framework to foster awareness and knowledge regarding the health aspects of climate change. Larger programs and schools should have access to expertise and resources to adopt all tiers of the framework to provide students a range of opportunities to learn about climate and health.

While this framework focuses on graduate education, it can be adapted and tailored for integration of climate change into undergraduate education including standalone public health bachelor’s programs. Small local health departments are less likely to employ public health professionals such as epidemiologists and statisticians that have graduate degrees (19). Therefore, building local public health capacity will require training students at both undergraduate and graduate levels on the complexities associated with climate change. Thus, we advocate for the adoption of this framework to train students regardless of degree (e.g., undergraduate or graduate) or health field focus (e.g., public health, medicine, nursing) as well as for continuing workforce education. Finally, we encourage the integration of climate change into existing accreditation criteria and credentialing mechanisms.

## Data availability statement

The raw data supporting the conclusions of this article will be made available by the authors, without undue reservation.

## Author contributions

MA: analyzed the data. All authors conceived and designed the study, and contributed to the manuscript.

## Funding

This work was supported in part by the National Oceanographic and Atmospheric Administration (NOAA), Climate Assessment for the Southwest (CLIMAS), the Arizona Department of Health Services, and the Centers for Disease Control and Prevention.

## Conflict of interest

The authors declare that the research was conducted in the absence of any commercial or financial relationships that could be construed as a potential conflict of interest.

## Publisher’s note

All claims expressed in this article are solely those of the authors and do not necessarily represent those of their affiliated organizations, or those of the publisher, the editors and the reviewers. Any product that may be evaluated in this article, or claim that may be made by its manufacturer, is not guaranteed or endorsed by the publisher.
